# Very Low Energy Ketogenic Therapy (VLEKT), Not Only a Price Matter: A Comparison Study on Average Costs of VLEKT with Fresh Foods and Replacement Meals

**DOI:** 10.3390/nu17030422

**Published:** 2025-01-24

**Authors:** Giuseppe Annunziata, Ludovica Verde, Andrea Falco, Silvia Savastano, Annamaria Colao, Giovanna Muscogiuri, Luigi Barrea

**Affiliations:** 1 Facoltà di Scienze Umane, della Formazione e dello Sport, Università Telematica Pegaso, Via Porzio, Centro Direzionale, Isola F2, 80143 Naples, Italy; giuseppe.annunziata@unipegaso.it; 2 Department of Public Health, University of Naples Federico II, Via Sergio Pansini 5, 80131 Naples, Italy; ludovica.verde@unina.it; 3 Department of Health Sciences, European University of Madrid, 28670 Madrid, Spain; 4 Unit of Endocrinology, Diabetology, Andrology and Nutrition, DAI of Endocrinology, Diabetology, Andrology, Centro Italiano per la Cura e il Benessere del Paziente con Obesità (C.I.B.O), Azienda Ospedaliera Universitaria Federico II, Via Sergio Pansini 5, 80131 Naples, Italygiovanna.muscogiuri@unina.it (G.M.); 5 Unit of Endocrinology, Dipartimento di Medicina Clinica e Chirurgia, Federico II University Medical School of Naples, Via Sergio Pansini 5, 80131 Naples, Italy; 6 Cattedra Unesco “Educazione Alla Salute e Allo Sviluppo Sostenibile”, University Federico II, 80131 Naples, Italy; 7 Dipartimento Psicologia e Scienze della Salute, Università Telematica Pegaso, Centro Direzionale Isola F2, Via Porzio, 80143 Naples, Italy

**Keywords:** very low calorie ketogenic diet (VLCKD), very low energy ketogenic therapy (VLEKT), obesity, costs, replacement meals, diet

## Abstract

Background: Obesity is constantly growing worldwide, representing a serious concern also for healthcare costs. Current anti-obesity pharmacological strategies, although effective, represent a significant cost for the patient. Similarly, very low energy ketogenic therapy (VLEKT) protocols with replacement meals also have high costs. Objectives: The objective of this study was to estimate the average costs of a VLEKT protocol with replacement meals compared with those of isocaloric diets with fresh foods. Methods: VLEKTs with replacement meals and fresh foods were developed considering an ideal young woman and man with grade II obesity (BMI ≥ 35.0 kg/m^2^). The costs of the individual fresh foods were extrapolated from official Italian databases. The costs of replacement meals were obtained by consulting the catalogs of three companies specialized in VLEKTs operating in Italy. Results: On a monthly basis, VLEKT with fresh food had an average cost of EUR 253.44 and EUR 295.67, while VLEKT with replacement meals had an average cost of EUR 434.91 and EUR 535.99, for the woman and man, respectively. Conclusions: Although more expensive than a common diet, VLEKT should be seen not only as a dietary method for losing weight, but as a non-pharmacological, medicalized nutritional therapy, useful for managing various conditions, even those not directly related to obesity. Like a drug therapy, VLEKT requires the use of specific products that entail a higher cost, to be borne by the patient, but whose benefits should be emphasized, which go beyond weight loss and concern general health, thus considering them as a targeted nutritional strategy.

## 1. Introduction

The growing incidence of obesity globally is a serious concern, increasingly evolving into a true health emergency of pandemic proportions. This condition has a significant impact on national healthcare costs [1], including childhood obesity [2], given the coexistence of endocrine–metabolic alterations. Parallelly, it has been estimated that in the upcoming years, increases in healthcare costs of cancer may be attributable to excess body weight [3]. Despite the availability of various preventive and therapeutic strategies, including both pharmacological and non-pharmacological approaches, obesity prevalence continues to rise, indicating significant gaps in the effectiveness and accessibility of current interventions. In particular, the prevalence of obesity continues to rise steadily, despite the considerable efforts of research, which has directed considerable resources into the development of new preventive and therapeutic strategies, both pharmacological and non-pharmacological (i.e., targeted dietary interventions). These interventions, however, also represent a considerable cost. As an example, it was recently estimated that the net price of a monthly supply of Glucagon-like peptide-1 receptor agonists, an effective anti-obesity drug, ranges from USD 717 to USD 761 (about EUR 678 and EUR 720) [4]. Most importantly, these costs are borne by the patient.

Among the main non-pharmacological interventions, the ketogenic diet, and in particular the very low calorie ketogenic diet (VLCKD), is now widely recognized as a valid tool in the management and non-pharmacological treatment of obesity and its complications [5]. VLCKD, like the other types of ketogenic diets, is characterized by a carbohydrate intake of no more than 30–50 g *per* day, but differs from the others in that its calorie intake does not exceed 800 kcal *per* day. While its effectiveness in weight loss has been established, the broader relevance of VLCKD as a therapeutic tool lies in its additional metabolic and clinical benefits, such as improved glycemic control, reduced inflammation, and enhanced metabolic flexibility, which are not as comprehensively achieved with other dietary therapies [6]. Glucose restriction leads to a reduction in blood glucose levels, which, in turn, leads to a decrease in insulin secretion and an increase in glucagon production. This hormonal alteration is a stimulus for the release of triglycerides from adipocytes. The triglycerides, once hydrolyzed, release free fatty acids, which, bound to albumin, are transported via the blood stream to the liver, where they are converted into ketone bodies. The latter provide an alternative energy source for organs that are not involved in the production of ketone bodies, such as the muscles and brain [7]. This peculiar dietary manipulation induces a metabolic condition referred to as ‘nutritional ketosis’ (NK), distinct from diabetic ketoacidosis (DKA), which is a pathological state. NK, in fact, is characterized by blood ketone concentrations between 0.5 and 3 mg/dL, unlike DKA, in which the plasma ketone concentration is five to ten times higher. An interesting aspect of NK is the phenomenon of ‘keto-adaptation’, which occurs after a few weeks and corresponds to the ability of cells to adapt to using ketone bodies as their main source of energy instead of glucose. This process manifests itself through the activation of genes responsible for the synthesis of metabolic enzymes, resulting in increased mitochondrial density in oxidative tissues, such as the muscles and brain. Furthermore, during NK, the production of endogenous insulin helps prevent the onset of DKA by maintaining a stable metabolic balance, with normal blood glucose values and a physiological pH [7].

As far back as 2013, Paoli and colleagues published an inspiring review of the literature in which it emerged that weight loss was only one of the proven or, at the time, confirming effects of this dietary regimen, which, therefore, was beginning to acquire a more therapeutic connotation than merely being nutritional [8]. Very recent studies have confirmed Paoli’s insights by demonstrating, among others, the efficacy of VLCKD with replacement meals in improving clinical outcomes in patients with skin diseases [9] and mild kidney failure [10]. Although in all these studies the lowest common denominators, in terms of the mechanisms of action for the effects observed, were weight loss and a reduction in fat mass, further effects emerged, such as an improvement in intestinal dysbiosis, inflammation, and oxidative stress, as well as a positive effect on the sympathetic nervous system and hypothalamic–pituitary–adrenal axis [11], which open up new frontiers regarding the efficacy and applicability of this nutritional approach in other clinical contexts.

Overall, this evidence further underlines the therapeutic role of VLCKD, beyond simple weight loss, so much so that this nutritional approach has recently been officially renamed very low energy ketogenic therapy (VLEKT) [12], stressing two fundamental concepts: (1) the need to maintain a very low calorie intake (and not just carbohydrate, which is the prerogative of all ketogenic diets) and (2) the need to consider this approach not just as a diet, but as a true non-pharmacological nutritional therapy [12]. Conceived as such, therefore, VLEKT must follow the official guidelines which, in addition to establishing its prescriptive appropriateness according to the subject’s characteristics [5], also stipulate its ‘dosage and posology’ in terms of nutritional intake. In this sense, according to the most recent indications, in order to be effective in its therapeutic significance, VLEKT should be formulated according to the KeNuT multiphasic protocol which, among other things, envisages the use of replacement meals capable not only of ensuring a varied diet in terms of protein sources, but also of maintaining the quantitative control of other macronutrients, such as lipids, necessary to ensure the hypocaloric feature [7].

The use of replacement meals (which are marketed in Italy by specific companies specialized in ketogenic diets) clearly raises some concerns. Leaving aside the question of the safety of these products, which are in fact foodstuffs or food supplements and therefore subject to current legislation on the subject [13], the main concern is their costs. Replacement meals, in fact, are produced and distributed by specific companies and are, therefore, not freely available in ordinary supermarkets and can only be purchased on the recommendation of the health professional (physician or nutritionist) who has ascertained the feasibility of VLEKT for the patient. Given these premises, therefore, it is clear how the price of these products can be relatively high, resulting in a higher overall cost of the nutritional plan than that of other non-ketogenic plans or plans that are still ketogenic but elaborated without the use of replacement meals.

Data on the costs of VLEKT elaborated according to the KeNuT protocol are currently not available in the literature. Therefore, this study aimed to fill this gap by comparing the costs of two types of VLEKT: one based on fresh foods and the other utilizing commercially available replacement meals. This analysis is particularly relevant in light of the growing interest in VLEKT as a structured, therapeutic nutritional approach supported by clinical guidelines.

## 2. Materials and Methods

This study was designed to compare the costs associated with two types of VLEKT: one based on fresh foods and the other utilizing commercially available replacement meals.

The dataset contains price information for various food items categorized into groups such as meat, cheese, fish, vegetables, fruits, condiments, and miscellaneous products. Prices are primarily provided in kilograms (kg) or liters (L), representing standard units for bulk food items. For each item, a price per unit is listed, with some prices adjusted for inflation, reflecting the real value by correcting for inflation using official indices. The inflation adjustments are based on data from the Italian National Institute of Statistics (ISTAT) [14]. Other sources of data (e.g., CIAL.it) may be used when ISTAT references are unavailable. Missing data for certain items may reflect either seasonal price fluctuations or the unavailability of pricing information at the time of data collection.

The prices of ketogenic products were collected from three main companies operating in Italy in the ketogenic diet sector, in particular marketing products for VLEKT diet plans.

Statistical analysis was performed using descriptive statistics (mean ± standard deviation) for nutritional values and costs.

## 3. Results

In order to elaborate hypothetical VLEKT nutrition plans compatible with the real setting, we considered two ideal profiles of potential subjects (one man and one woman) who are young, with grade II obesity (Table 1).

The VLEKT regimens were formulated according to the KeNuT multiphasic protocol [7], ensuring both regimens provided similar caloric intakes and macronutrient distributions (Table 2). While both VLEKT approaches maintain similar energy and protein levels, replacement meals provide higher carbohydrate and fiber content, along with lower saturated fatty acids (SFAs), compared to fresh foods. Notably, the eicosapentaenoic acid (EPA) and docosahexaenoic acid (DHA) content is substantially lower in replacement meal-based plans.

The VLEKT plan with fresh foods included a weekly meal schedule for both men and women, consisting of breakfast, snacks, lunch, and dinner with whole food ingredients such as lean meats, fish, eggs, and low-carbohydrate vegetables. Extra virgin olive oil (EVOO) was used for cooking and added to meals to provide healthy fats (Table 3). In contrast, the VLEKT plan with replacement meals involved the use of commercially available ketogenic products, such as hot drinks, yogurt, pasta, rice, and cream. These products were selected to meet the nutritional needs of a ketogenic diet while providing controlled calorie intake. Also, for the VLEKT plan with replacement meals, EVOO was used for cooking and considered as the unique fat source (Table 4).

The average costs of fresh food (obtained from ISTAT and CIAL.it data) and replacement meals (extrapolated from the catalogs of the three companies taken as reference) used for the elaboration of the VLEKT plans are shown in Table 5 and Table 6, respectively.

The total costs of both VLEKT plans were calculated on a daily, weekly, and monthly basis, as reported in Table 7.

## 4. Discussion

This study focused on a comparison of the costs of a VLEKT elaborated with replacement meals *versus* one elaborated with fresh foods. In order to calculate the costs of replacement meals, the prices reported in the informative materials of three companies distributing replacement meals, operating in Italy, were considered. The comparison showed that, overall, VLEKT with replacement meals is more expensive than VLEKT with fresh foods.

If purely economic issues are considered, the result was expected. Suffice it to say that replacement meals are subject to complex production processes that are different, or non-existent, for many fresh foods, which may justify their higher cost.

These products, indeed, are subject to prescription by health professionals and are not available in large-scale distribution, but sold exclusively by specialized companies. These additional aspects justify their higher cost compared to foods bought in supermarkets. Replacement meals, thus, are not to be understood as foods consumed daily throughout life, but as specific products required to adhere to a nutritional protocol with therapeutic purposes (such as VLEKT) of variable, but always time-limited, duration. In this sense, according to current guidelines, the ketogenic phases of the KeNuT protocol can last between 8 and 12 weeks [7]. This, of course, also affects the total costs of VLEKT, whether elaborated with replacement meals or fresh food.

From this point of view, the concept of indispensability that binds replacement meals to a correct elaboration of the VLEKT lies in the added value that these products can have.

The use of replacement meals for VLEKT, in fact, has a number of advantages that we can describe as ‘quantitative’ and ‘qualitative’. From a quantitative point of view, in fact, replacement meals have a well-controlled nutritional composition containing, in general, about 15–18 g of high-biological-value proteins, no more than 4 g of fats, and, obviously, a very low carbohydrate intake (no more than 3.5 g of available carbohydrates) [7]. This specific composition of most replacement meals allows for an optimal micro- and macronutrient intake, which, among other things, is controlled by the availability of these products in single-serving portions [7]. It is universally known, in fact, that regardless of its type, the elaboration of a nutritional plan with weights and portion sizes of individual foods is a mere theoretical exercise based on theoretical assumptions (e.g., the estimated average amount of macronutrients present in a specific food), which cannot always reflect the real-life setting. The official food composition databases, in fact, contain data from suppliers (e.g., USDA in the USA [15]), experimental data on more widely consumed products (e.g., CREA in Italy [16]), or data from selected European countries (e.g., EFSA in Europe [17]). In a generalized sense, therefore, these data should be regarded as estimates, which are highly reliable but which, in any case, may deviate from the actual composition of the specific food that the consumer buys and consumes. Considering this aspect, it is clear that, apart from protein and carbohydrates, which could be macroscopically controlled in some way, in the context of a ketogenic diet, the precisely controlled lipid intake is fundamental, as it represents the ‘needle of the scales’ for managing calorie intake, which, in the case of VLEKT is kept very low precisely thanks to this macronutrient (< 20–30 g/day) [7]. In the VLEKT examples, we have proposed for the woman, in fact, the amount of lipids in the version with replacement meals is about 9–10 g daily lower, thus resulting in a theoretical change of about 100 kcal. At the same time, this aspect also allows greater overall control of the daily caloric intake, as the patient is not required to control food weight and portion sizes. This is an important advantage, given the tendency to overestimate the calories of foods [18], and it also allows increased dietary compliance from the patient [19]. With the use of replacement meals, compliance is further increased by their ability to reduce the hedonic drive towards food due to an attenuation of the sensory stimuli given by their intake [7], stimuli which, if of a high magnitude, contribute to increasing caloric intake, predisposing to obesity [20]. Overall, these aspects translate into better weight loss results with the use of replacement meals, as previously reported in a meta-analysis [21], thus suggesting the use of these products as a therapeutic strategy, rather than a dietary alternative.

The concept of using replacement meals as a strategy is further reinforced by analyzing their qualitative advantages. Firstly, the replacement meals used for VLEKT contain proteins with a high biological value [7] which, when present in optimal quantities, help to preserve muscle mass during weight loss by maintaining constant insulin levels and promoting the release of growth hormone (GH) [22]. Furthermore, replacement meals make it possible to develop a nutritional plan that does not involve a high consumption of animal protein sources, with consequent advantages for the patient’s general health. As reported in a previous study, in fact, 45 days of VLEKT with animal protein, compared to a plan with replacement meals, led to a worsening of renal parameters (increased urea nitrogen and uric acid, and reduced estimated glomerular filtration rate, e-GFR). Nevertheless, the most interesting finding is the one concerning intestinal well-being. Indeed, an improvement in the *Firmicutes*/*Bacteroidetes* ratio was observed in the group following VLEKT with replacement meals. The authors, therefore, concluded that this latter dietary approach has the real advantage of improving both the metabolic profile and the composition of the gut microbiota [23], which is associated with the canonical benefits of nutritional ketosis, making this approach appropriate for subjects with obesity and associated comorbidities, in particular cardiovascular risk [7]. To confirm this, the results highlighted key nutritional differences between fresh food- and replacement meal-based VLEKT plans. Replacement meals provided higher fiber and carbohydrate content, with the latter remaining within ketogenic thresholds. This reflects the standardized formulation of replacement meals, which enhances satiety and supports gut health. Additionally, their lower SFA content may benefit patients with cardiovascular risk, aligning with their therapeutic goals. However, replacement meal-based plans showed significantly lower EPA and DHA levels, due to the absence of natural sources like fresh fish. This underscores the need for supplementation or dietary adjustments to ensure adequate omega-3 intake. Overall, these differences suggest that replacement meals offer greater precision and control, potentially improving adherence and outcomes, while emphasizing the importance of individualized nutritional planning. Another factor to consider is the seasonality of fresh foods, which can cause significant price fluctuations. For example, fruits, vegetables, and some animal proteins are cheaper during peak seasons and more expensive during off-seasons. These variations can impact the overall cost of a fresh food-based VLEKT. In contrast, replacement meals, with their standardized, pre-packaged servings, remain unaffected by such seasonal changes, offering more cost stability over time. While fresh foods provide nutritional variety, replacement meals may offer a more predictable cost structure, particularly in the face of seasonal price shifts.

Taken together, these observations make it possible to outline a cost–benefit ratio, which, although not statistically analyzed in this study, appears to be unequivocally in favor of the use of replacement meals for the elaboration of VLEKT.

The main strength of this study is that it is the first time that a cost comparison of VLEKT elaborated with replacement meals or fresh foods is reported. Similarly, there are some limitations such as having only considered data on average food costs in Italy, without considering those of other European or non-European countries where the use of replacement meals for VLEKT elaboration is widespread. Similarly, no sub-analysis was conducted for single Italian regions or macro-areas (South, Central and North Italy), where food costs may vary considerably. Although interesting and useful, the purpose of this study was to provide a general overview of price differences, without analyzing single variables that nevertheless remain significant. Further research along these lines could clarify these aspects by also highlighting possible variations in cost disparities in various parts of Italy and the world. Furthermore, this is a preliminary study aimed at highlighting the existence of a cost difference between these two types of VLEKT, considering only the population with obesity and thus in a very specific setting, but this represents the main population to which this nutritional therapy is prescribed. However, it is important to emphasize that VLEKT protocols are highly structured and standardized dietary strategies, grounded in well-established clinical guidelines [6]. This structure allows for a degree of translatability, even when the study is based on hypothetical individuals, as the underlying principles of the diet can be adapted to various demographic and clinical contexts (e.g., age, BMI, or metabolic needs) with appropriate adjustments. We are aware of the importance of assessing costs in other clinical settings in which, however, the nutritional characteristics of ketogenic therapy (e.g., energy intake) may vary, also affecting costs. Additionally, future research should incorporate real-world data to evaluate critical factors such as patient adherence, dietary satisfaction, and long-term metabolic outcomes. These elements are crucial for validating the practical application of VLEKT and for understanding how its benefits translate into routine clinical practice. For these reasons, starting from these preliminary observations, future studies should thoroughly investigate the effective costs incurred by different types of populations, based on dietary prescription and geographical location. Similarly, important factors such as possible differences in adherence and personal satisfaction with one or another type of VLEKT should be investigated. Furthermore, expanding the sample to include individuals with varying clinical profiles and comorbidities would strengthen the generalizability of the findings, providing a more comprehensive understanding of the cost–benefit balance of VLEKT across diverse patient populations.

## 5. Conclusions

The most recent evidence suggests that VLEKT should be considered not just as a mere dietary approach aimed at weight loss, but as a true non-pharmacological medicalized nutritional therapy with the aim of managing various pathological conditions that may or may not be closely linked to obesity. From this point of view, in the same way as a pharmacological therapy, VLEKT requires the use of specific products which, being a dietary approach, are represented by replacement meals. Again, like pharmacological therapies, these products used for VLEKT, besides representing an objective advantage over fresh food, also have a higher cost, which, as is the case with many drugs, is now borne by the patient. Without prejudice towards the (perhaps utopian, but plausible) need for national and international legislative action to regulate the expenditure for these products to be borne by the National Health System, at least for certain classes of patients, there is a need for the healthcare professional prescribing VLEKT to carefully inform the patient about the cost–benefit ratio of using replacement meals. The patient should be informed of the benefits of using these products instead of ordinary food in the context of VLEKT, benefits that go beyond just weight loss and involve key aspects of global health. In this scenario, therefore, the healthcare professional and the patient must view replacement meals as a nutritional strategy and not as a dietary alternative, aimed at optimizing the effects of therapy and maintaining health status.

## Figures and Tables

**Table 1:** Ideal man and woman for VLEKT prescription.

Parameter	Man	Woman
Age (years)	35	35
Height (m)	1.75	1.65
Weight (kg)	113	100
BMI (kg/m^2^)	36.9	36.7
Ideal weight (kg)	67	60
Ideal BMI (kg/m^2^)	22	22

Abbreviation: body mass index, BMI.

**Table 2:** Average nutritional values of VLEKTs with fresh foods and replacement meals.

	VLEKT Woman		VLEKT Man	
	Fresh Foods	Replacement Meals	Fresh Foods	Replacement Meals
Energy (kcal)	740 ± 39.37	698.95 ± 34.61	735.60 ± 60.26	779.20 ± 42.38
Protein (g) and (%)	70.20 ± 7.13 (40.75)	69.84 ± 4.86 (43.20)	94.30 ± 13.44 (52.56)	86.70 ± 6.31 (47.34)
Carbohydrates (g) and (%)	14.70 ± 3.40 (8.01)	22.30 ± 6.24 (12.86)	18.10 ± 2.06 (9.5)	24.89 ± 7.39 (12.65)
Sugars (g)	13.00 ± 2.32	10.74 ± 2.06	15.90 ± 2.74	12.19 ± 2.97
Fiber (g)	6.20 ± 1.93	10.89 ± 6.39	7.80 ± 1.30	11.65 ± 7.28
Lipids (g) and (%)	39.30 ± 4.81 (51.24)	31.50 ± 1.57 (43.94)	30.30 ± 9.44 (37.98)	32.48 ± 1.84 (40.01)
SFA (g)	13.10 ± 1.39	6.06 ± 1.62	7.90 ± 4.81	6.49 ± 1.93
EPA + DHA (g)	1.60 ± 15.51	0.07 ± 0.12	1.80 ± 0.64	0.08 ± 0.12

Abbreviations: very low energy ketogenic therapy, VLEKT; saturated fatty acid, SFA; eicosapentaenoic acid, EPA; docosahexaenoic acid, DHA.

**Table 3:** Weekly VLEKT plan with fresh foods.

Meal	Monday	Tuesday	Wednesday	Thursday	Friday	Saturday	Sunday
**VLEKT woman**							
Breakfast	5% Greek yogurt (100 g) + dark chocolate (5 g)	5% Greek yogurt (100 g) + dark chocolate (5 g)	5% Greek yogurt (100 g) + dark chocolate (5 g)	5% Greek yogurt (100 g) + dark chocolate (5 g)	5% Greek yogurt (100 g) + dark chocolate (5 g)	5% Greek yogurt (100 g) + dark chocolate (5 g)	5% Greek yogurt (100 g) + dark chocolate (5 g)
Snack	Walnuts (5 g)	Almonds (5 g)	Walnuts(5 g)	Almonds (5 g)	Walnuts (5 g)	Almonds (5 g)	Walnuts (5 g)
Lunch	Mackerel (130 g) + courgettes (200 g) + EVOO (5 g)	Squid (200 g) + asparagus (200 g) + EVOO (5 g)	Cod (150 g) + lettuce (100 g) + EVOO (5 g)	Bresaola (140 g) + cucumber (200 g) + EVOO (5 g)	Squid (200 g) + asparagus (200 g) + EVOO (5 g)	Salmon (150 g) + courgettes (200 g) + EVOO (5 g)	Tuna (100 g) + peppers (200 g) + EVOO (5 g)
Dinner	Rabbit (150 g) + cucumber (200 g) + EVOO (5 g)	Eggs (2 units) + spinach (200 g) + EVOO (5 g)	Pork steak (150 g) + cauliflower (150 g) + EVOO (5 g)	Parmigiano cheese (30 g) + peppers (200 g) + EVOO (5 g)	Rabbit (150 g) + spinach (200 g) + EVOO (5 g)	Calf (150 g) + cauliflower (150 g) + EVOO (5 g)	Chicken (200 g) + lettuce (100 g) + EVOO (5 g)
**VLEKT man**							
Breakfast	0% Greek yogurt (200 g)	0% Greek yogurt (200 g)	0% Greek yogurt (200 g)	0% Greek yogurt (200 g)	0% Greek yogurt (200 g)	0% Greek yogurt (200 g)	0% Greek yogurt (200 g)
Snack	Walnuts (5 g)	Almonds (5 g)	Walnuts (5 g)	Almonds (5 g)	Walnuts (5 g)	Almonds (5 g)	Walnuts (5 g)
Lunch	Tuna (150 g) + spinach (200 g) + EVOO (5 g)	Salmon (150 g) + courgettes (200 g) + EVOO (5 g)	Squid (300 g) + asparagus (200 g) + EVOO (5 g)	Bresaola (140 g) + fennels (200 g) + EVOO (5 g)	Cod (250 g) + lettuce (100 g) + EVOO (5 g)	Turkey (150 g) + asparagus (200 g) + EVOO (5 g)	Mackerel (130 g) + courgettes (200 g) + EVOO (5 g)
Snack	Dark chocolate (5 g)	Black olives (15 g)	Dark chocolate (5 g)	Black olives (15 g)	Dark chocolate (5 g)	Black olives (15 g)	Dark chocolate (5 g)
Dinner	Parmigiano cheese (50 g) + lettuce (100 g) + EVOO (5 g)	Calf (200 g) + eggplants (200 g) + EVOO (5 g)	Rabbit (150 g) + courgettes (200 g) + EVOO (5 g)	Cod (250 g) + spinach (200 g) + EVOO (5 g)	Turkey (150 g) + fennels (200 g) + EVOO (5 g)	Eggs (2 units) + spinach (200 g) + EVOO (5 g)	Chicken (200 g) + fennels (200 g) + EVOO (5 g)

Abbreviations: very low energy ketogenic therapy, VLEKT; extra virgin olive oil, EVOO.

**Table 4:** Example of VLEKT with replacement meals.

Meal	VLEKT Woman	VLEKT Man
Breakfast	Ketogenic hot drink	Ketogenic hot drink
Snack	Ketogenic yogurt or dessert or mousse	Ketogenic yogurt or dessert or mousse
Lunch	Ketogenic pasta or rice or bread + selected vegetables (100 or 200 g) + EVOO (15 g)	Ketogenic pasta or rice or bread + selected vegetables (100 or 200 g) + EVOO (15 g)
Snack	-	Ketogenic yogurt or dessert or mousse
Dinner	Ketogenic cream or omelette + selected vegetables (100 or 200 g) + EVOO (10 g)	Ketogenic cream or omelette + selected vegetables (100 or 200 g) + EVOO (10 g)

Abbreviations: very low energy ketogenic therapy, VLEKT; extra virgin olive oil, EVOO.

**Table 5:** Average costs of food included in the VLEKT plans.

Food	Unit of Measure	Reference Value	Cost (EUR)
Meat			
Beef	kg	1	21.48
Bresaola	kg	1	50.60
Calf	kg	1	22.49
Chicken	kg	1	9.49
Ham	kg	1	31.90
Pork	kg	1	13.70
Rabbit	kg	1	14.49
Turkey	kg	1	15.49
Dairy products			
Mozzarella cheese	kg	1	13.95
Parmigiano cheese	kg	1	28.60
Greek yogurt	kg	1	2.03
Fish			
Cod	kg	1	26.30
Mackerel	kg	1	32.50
Salmon	kg	1	30.00
Squid	kg	1	20.00
Tuna	kg	1	27.50
Vegetables			
Asparagus	kg	1	2.50
Cauliflower	kg	1	1.60
Courgette	kg	1	3.31
Cucumber	kg	1	0.62
Eggplant	kg	1	2.35
Fennel	kg	1	1.19
Lettuce	kg	1	0.87
Pepper	kg	1	3.98
Spinach	kg	1	0.71
Seasoning			
EVOO	l	1	8.60
Other			
Almonds	kg	1	19.90
Black olives	kg	1	12.93
Dark chocolate	kg	1	10.90
Eggs	kg	1	6.26
Walnuts	kg	1	12.96

Abbreviations: euro, EUR; extra virgin olive oil, EVOO.

**Table 6:** Costs of single replacement meals selected for the VLEKT plans.

Companies	Meal	Replacement Meals	Cost *per* Unit (EUR)
1	Breakfast	Hot drink	3.43
	Snack	Yogurt	3.43
		Dessert	3.43
		Mousse	3.43
	Lunch	Pasta	3.43
		Rice	3.5
	Dinner	Cream	3.43
		Omelette	3.43
2	Breakfast	Hot drink	3.5
	Snack	Yogurt	3.5
		Dessert	3.5
	Lunch	Toasted bread	3.3
		Flat bread	2.25
		Cereal bread	3
		Bread	2.2
	Dinner	Cream	3.5
		Omelette	3.5
3	Breakfast	Hot drink	3.9
	Snack	Dessert	3.9
	Lunch	Pasta	4.85
	Dinner	Cream	4.1
		Omelette	4.1
Average	Breakfast		3.61 ± 0.25
	Snack		3.53 ± 0.18
	Lunch		3.22 ± 0.90
	Dinner		3.68 ± 0.33

Abbreviations: euro, EUR.

**Table 7:** Total costs of VLEKT plans with fresh foods and replacement meals.

Diet	Total Costs (EUR)		
	Day	Week	Month (4 wks)
Fresh foods			
VLEKT woman	9.05 ± 1.95	63.36	253.44
VLEKT man	10.56 ± 6.69	73.92	295.67
Replacement meals			
VLEKT woman	15.53 ± 1.89	108.73 ± 13.24	434.91 ± 52.94
VLEKT man	19.14 ± 2.14	134 ± 14.95	535.99 ± 59.82

Abbreviation: euro, EUR; weeks, wks; very low energy ketogenic therapy, VLEKT.

## Data Availability

The original contributions presented in the study are included in the article, further inquiries can be directed to the corresponding author.
